# Anthocyanin-rich extract from black beans exerts anti-diabetic effects in rats through a multi-genomic mode of action in adipose tissue

**DOI:** 10.3389/fnut.2022.1019259

**Published:** 2022-11-14

**Authors:** Karla Damián-Medina, Dragan Milenkovic, Yolanda Salinas-Moreno, Karla Fabiola Corral-Jara, Luis Figueroa-Yáñez, Erika Marino-Marmolejo, Eugenia Lugo-Cervantes

**Affiliations:** ^1^Food Technology Unit, Center for Research and Assistance in Technology and Design of the State of Jalisco, A.C. (CIATEJ), Guadalajara, Jalisco, Mexico; ^2^Department of Nutrition, University of California, Davis, Davis, CA, United States; ^3^National Institute of Forestry, Agriculture and Livestock Research (INIFAP), Guadalajara, Jalisco, Mexico; ^4^Université Clermont Auvergne, INRAE, UNH, Clermont-Ferrand, France; ^5^Industrial Biotechnology Unit, Center for Research and Assistance in Technology and Design of the State of Jalisco, A.C. (CIATEJ), Guadalajara, Jalisco, Mexico; ^6^Medical and Pharmaceutical Biotechnology Unit, Center for Research and Assistance in Technology and Design of the State of Jalisco, A.C. (CIATEJ), Guadalajara, Jalisco, Mexico

**Keywords:** black beans, type 2 diabetes mellitus, polyphenols, anthocyanins, adipose tissue, multi-genomics, nutrigenomics

## Abstract

Black beans (BB) are an important source of a range of plant bioactive compounds including polyphenols, particularly anthocyanins. Several studies support that consumption of BB is associated with health benefits, including prevention of type 2 diabetes mellitus (T2DM). However, molecular mechanisms underlying the potential health properties of BB on adipose tissue (AT) are still largely unknown. The purpose of this study was to investigate multi-genomic effects of BB intake and identify regulatory networks potentially mediating T2DM on AT. Male Wistar diabetic rats consumed an anthocyanin-rich black bean extract for 5 weeks. Global gene expression from AT, protein coding and non-coding RNA profiles were determined using RNAseq. Biological function analyses were performed using a variety of bioinformatic tools. The evaluation of global gene expression profiles exhibited significant change following BB consumption with 406 significantly differentially expressed genes, 33 miRNA and 39 lncRNA and 3 snRNA. Functional analyses indicated that these genes play an important role in regulation of PI3K signaling, NIN/NF-kB signaling, insulin secretion, and endoplasmic reticulum (ER) organization. Interestingly, transcription factors such as GATA2, or POU2AF1 demonstrated to modulate their activity by BB extract by direct interaction with polyphenol metabolites, or by interactions with cell signaling proteins, like PKB, AKT or PI3K, that could control transcription factor activity and as a result impact on adipogenesis regulation. Therefore, the constant consumption of an anthocyanin-rich black bean extract may have anti-diabetic protective effects by modulating gene expression, resulting in a promising alternative for T2DM patients.

## Introduction

According with the International Diabetes Federation (IDF), the prevalence of people with T2DM raised from 108 million to 537 million in the last 40 years. T2DM is a disease caused by the incapacity of the cells to respond to insulin effects. This disease is strongly linked with obesity resulting from the excess of body weight and sedentarism (1). Obesity is considered a complex chronic progressive disease defined as abnormal or excessive adipose tissue accumulation caused by an imbalance of energy intake and energy expenditure that leads to mild, chronic, systemic inflammation. Diet is the main risk factor that contributes to obesity (2). In a cross-sectional study from 2015 in Canadian adults, it was reported that ultra-processed food consumption, which contributed to 24-73% of total daily energy intake, was associated with higher prevalence of obesity in the studied population (3). It was also reported that high fat diets and elevated intake of red meat (4, 5) as well as high sugar intake (6) impairs health causing obesity and increase the risk of non-communicable diseases (NCD’s) such as type 2 diabetes mellitus (T2DM), dementia, myocardial infarction, stroke, hypertension, fatty liver disease and cancer (7). In general, obesity is associated with a lower life quality and expectancy by an estimated of 5-20 years depending on the severity and NCD’s (8).

Adipose tissue has a crucial role on systemic energy balance (9), playing an essential role in maintaining lipid and glucose homeostasis. The fat stored as triglycerides tends to accumulate in the subcutaneous and visceral depots, incrementing their size and producing hypertrophy, hyperplasia, and systemic metabolic dysfunction (10). The main mechanism believed to link obesity with T2DM is insulin resistance derived from adipose tissue accompanied by impaired insulin secretion by β-cells in the pancreas. Free fatty acids stimulate NF-κB and P38 MAPK signaling pathway through MyD88 and TRIF-mediated downstream pathways with a subsequent activation of TLR4 expression in adipocytes and macrophages, increasing ER stress and producing ROS, promoting the secretion of pro-inflammatory cytokines, causing the initial step of low-grade systemic inflammation (11). The pro-inflammatory adipokines secreted by adipose tissue are monocyte chemotactic protein-1 (MCP-1), tumor necrosis factor α (TNF-α), interleukin 1-β (IL-1β), and interleukin-6 (IL6) (12). The potential cellular mechanisms of obesity-induced insulin resistance start with increased systemic TNF-α which stimulates the activity of IKK, p38 MAPK, JNK, and PKC, insulin receptor substrate (IRS); impairing tyrosine phosphorylation and increasing the risk of insulin resistance in adipose tissues, muscles, and liver (13).

Lifestyle modifications with controlled calorie intake, healthy diet and increased physical activity are considered the fundamental basis of a successful treatment (2). Higher protein intake, foods with a low glycemic index and lower fat consumption may help diabetic patients (9). Moreover, recent studies suggest that the intake of fresh fruits and vegetables is related with a lower incidence of T2DM. In a randomized controlled trial, it has been explained and demonstrated how diets rich in fruits and vegetables can improve blood glucose and insulin secretion (14–17). Furthermore, studies also indicate that bioactive compounds present in fruits, vegetables, spices, legumes, edible flowers, mushrooms, and medicinal plants, are prospective candidates for the prevention and the control of T2DM (18, 19).

Fruits and vegetables are rich sources of bioactive compounds, particularly polyphenols, exerting numerous positive human health effects. Polyphenols are plant secondary metabolites that act as a defense against pathogens, diseases, predators, ultraviolet radiation, parasites, and oxidants (20). Polyphenols are widely used in the food industry as a natural antioxidant ingredient in foods by their effects on preventing lipid oxidation and oxidative rancidity (21). Polyphenols are classified as flavonoids and non-flavonoids; flavonoids are divided in 12 groups including, flavonols, flavanols, flavons, flavan-3-ols, anthocyanins, flavanones, isoflavones, and dihydrochalcone. Non-flavonoids include phenolic acids, lignans and stilbenes. Following intake of polyphenols, they undergo important metabolism (22), which start in the enterocytes of small and large intestines before being absorbed into the circulation. Once in the hepatocytes, the hydroxyl group in the flavonoids undertake glucuronidation, methylation and sulfation to be available to enter in blood circulation. The next step is their flux to all organs and finally their elimination through urine. In the large intestine, the colonic microbiota will produce an extensive breakdown of the original polyphenolic structure into a phenolic metabolites with low-molecular-weight, this will produce a better absorbability of the compounds, and its passage to the secondary phase metabolism (23).

Anthocyanins are hydrophilic pigments contained in red, violet, and blue colors of fruits and vegetables. Most abundant anthocyanins are glycosylated forms of cyanidin, delphinidin, malvidin, peonidin, petunidin, and pelargonidin. Anthocyanins are commonly linked to a sugar molecule, frequently glucose, nevertheless, rhamnose, galactose, and rutinose can also be present. Anthocyanins play an important role in obesity and diabetes prevention. Different studies have suggested that, once absorbed, anthocyanins can positively modulate GLUT4 in skeletal muscle and adipose tissue; other authors conclude that these phytochemicals may affect gastrointestinal microbiota and impact the host health (24, 25). One of the foods rich in anthocyanins are BB. The seed color of BB is determined mainly by the presence of anthocyanins, and condensed tannins (proanthocyanidins). As one of the main flavonoid groups found in BB, anthocyanins have been shown to determine the color of the seed coats, but also demonstrated to be biologically active and have potential health properties (26).

BB have been suggested to contribute to the treatment of T2DM. Anthocyanins from BB have a strong antioxidant ability to arrest free radicals along with anti-inflammatory activity (27). We previously showed through an *in silico* perspective, that polyphenols found in BB, particularly anthocyanins, could modulate the activity of proteins involved in different mechanisms of T2DM pathways (28). Molecular docking results highlighted that cyanidin 3-glucoside, delphinidin 3-glucoside, and petunidin 3-glucoside had a stronger affinity for 11β-HS, GFAT, PPARG, PTP, RTKs, and PTP, proteins related with mechanisms that can regulate different biomarkers linked to inflammation, insulin resistance, oxidative stress, glucose and lipid metabolism, insulin secretion, and carbohydrate absorption (28). The mode of action underlying the anthocyanin biological effects has been attributed only to their direct antioxidant properties. However in recent years, it has been shown that these bioactive compounds exert more complex molecular mechanisms of action, including modulation of gene expression, cell signaling, or DNA methylation (29). Most of the studies performed used targeted approaches so global mechanisms are still little known.

Taken together, the aim of this study was to characterize anti-diabetic properties of an anthocyanin-rich extract from BB and decipher, using RNAseq approach, molecular mechanisms of action of these bioactive compounds on adipose tissue in a diabetic rat model.

## Materials and methods

### Extracts preparation

We previously reported the chemical composition of BB extract (28). Polyphenol extraction from BB was performed on recently harvested BB which were finely ground until obtaining a flour consistency. The flour was mixed with a solution of ethanol (99.9%) and hydrochloric acid (0.1%). The mix was stirred for 4 h at room temperature and covered from light. The extracts were centrifuged for 20 min at 13,000 rpm, the supernatant collected and evaporated at 38°C and 90 rpm until ethanol was completely removed. The extracts were stored at −20°C overnight and lyophilized during three days at −50°C and 250 mBar. The polyphenolic powders were conserved at 4°C until their use (28).

### Animal experiments

The animal experiments were approved by the Internal Committee of Care and Use of Laboratory Animals (CICUAL) of the Center for Research and Assistance in Technology and Design of the State of Jalisco, A.C., (CIATEJ A.C.) according with the Official Mexican Standard NOM-062-ZOO-1999, concerning the technical specifications for the production, care and use of laboratory animals and NOM-087-ECOL-SSA1-2002, related to the Management of Infectious Biological Hazardous Waste -RBPI. In addition, the Standard Bioterial Operation Procedure (UEP-PNO-BIO-001) and the Animal Experimentation Laboratory Regulations (REG-SM/BM-01) were applied. A total of 24 male Wistar rats around 150 ± 20g were purchased from Envigo RMS S.A de C.V. and placed in a SPF barrier environment under standard environmental conditions (temperature 25°C, relative humidity 60 ± 5%) under 12 h light/dark cycle, with free access to water and standard diet (Envigo Teklad T.2018S.15) during the acclimation stage. After 2 weeks of acclimatization, all the rats were randomly divided into three groups (*n* = 8), namely the HE (healthy group without treatment), BB (animals with induced T2DM treated with 260 mg/kg/day of black bean extract), and DB animals with induced T2DM without treatment (this group was provided with 1 ml/day of water via oral gavage). During the experiment the HE group was fed with standard diet, while BB and DB groups were fed with a high fat diet (HDF) (Envigo Teklad TD.06414) consisting of 42.7% carbohydrates, 42% lipids and 15.2% proteins. After the HFD feeding for 5 weeks, the rats were fasted for 12 h with unlimited access to water. Rats from HE group received an intraperitoneal injection of citrate buffer. Our T2DM induction is supported by the methodology reported by Skovsko, (30), where the prolonged administration of HFD combined with a single low dose of Streptozotocin produce a partial damage of pancreatic B-cells accompanied by lipotoxicity, glucolipotoxicity, insulin resistance, and hyperinsulinemia caused by HFD. Rats in BB and DB groups received an intraperitoneal injection of a single low dose of Streptozotocin, N-(Methylnitrosocarbamoyl)-α-D-glucosamine (STZ) (Sigma-Aldrich) solution (25 mg/kg) dissolved in sodium citrate buffer (0.1 mol/L, pH 4.5). The successful diabetes induction was confirmed with blood glucose measurements from the tail vein with a glucometer (Accu-Check Active^®^, Roche. Basel, Switzerland), all the measurements were taken during fasting and postprandial periods during 3 days after injection. Values of fasting glucose higher than 200 mg/dl indicated a successful establishment of T2DM rat model. The BB extract was diluted in 1ml of water and administrated by oral gavage for 31 days. The fasting glucose levels in blood were tested every 2 days per week from the tail vein. Water and food consumption were quantified every day and body weight was monitored once a week.

### Sample collection and preparation

At the end of the treatment period, the rats were anesthetized via intraperitoneal with 3% pentobarbital sodium (0.3 ml/100 g body weight). Blood samples were collected by cardiac puncture, the blood was centrifuged for 15 min at 13,000 rpm and the plasma was recovered and stored in aliquots of 500 μl at −20°C until use. Retroperitoneal adipose tissue was aliquoted, isolated, and washed with PBS and frozen immediately with liquid nitrogen. The frozen samples were stored at −80°C. The stored serum samples were thawed at 4°C. Insulin levels were quantified using the Rat Insulin ELISA kit RayBio^®^. Peachtree Corners, GA. The levels of total cholesterol, triglycerides, HDL-Cholesterol, and LDL-Cholesterol in the serum samples were measured with commercial kits for colorimetric assays. Tumor Necrosis Factor alpha (TNF-a) was quantified with ELISA kit (RayBio^®^ TNF. Peachtree Corners, GA) according to manufacturer protocols.

### RNA extraction and sequencing

RNA extraction from adipose tissue samples were carried out according to the protocol proposed in the RNeasy Lipid Tissue Mini Kit (Qiagen UK). The sequencing library was prepared by random fragmentation of the cDNA, followed by the 5 ‘and 3’ ligation adapters. Adapter-ligated fragments were amplified by PCR and visualized on agarose gels. Double stranded next generation sequencing RNAseq was commercially performed by MAcrogen (Seoul, Korea) on Illumina HiSeq4000 by triplicate in each group. All RNAseq data are available at GEO database under accession number GSE215903.

### Bioinformatic analysis

#### Differentially expressed genes

Pair-wise comparisons between biological conditions (DB, BB, and HE) were performed using T-test and Fold Change (FC). A correction for multiple testing was applied using the Benjamini–Hochberg procedure to control the false discovery rate (FDR). Probes with FDR-adjusted *P* > 0.05 were considered differentially expressed between conditions. Gene types of the differentially expressed genes (mRNA, miRNA, and lncRNA) were identified using ShinyGO v0.66^1^ (31).

#### Pathways and network analyses

Interaction networks were constructed with Cytoscape software, version 3.7.2.^2^ Gene ontology and interactions were identified using Metascape tool within Cytoscape^3^.

#### Protein-protein interactions

STRING software version 11.0^4^ was used for protein-protein interaction analyses, including physical and functional associations, network building and identification of proteins with highest number of interactions.

#### Database-predicted miRNA

Target genes of the identified miRNAs were searched with MIENTURNET^5^. Network-based visualization of miRNA-gene target enrichment was also performed with MIENTURNET (http://userver.bio.uniroma1.it/apps/mienturnet/).

#### Transcription factor analysis

The mail transcription factors which activity could be modulated by polyphenols were identified with bioinformatic tool Enrichr^6^. TRRUST and TRANSFAC databases were used to search for the potential transcription factors.

#### Docking analysis

We previously characterized the composition of BB extract by UPLC-ESI/qTOF/MS. We found that Delphinidin 3-glucoside, Petunidin 3-glucoside, and Malvidin 3-glucoside, are the three main anthocyanins in the extract (28). For this reason, we considered relevant to test the potential binding interactions between identified transcription factors, their regulatory cell signaling proteins, and anthocyanin metabolites by molecular docking using the SwissDock docking analysis tool^7^. Protein 3D structures were searched and downloaded in pdb. format from UniProt Data Bank^8^. Chemical structures of metabolites were downloaded from PubChem database^9^ and converted to MOL.2 format.

#### IncRNA target interaction analysis

We used the bioinformatic tool LncRRIsearch to identify potential targets of differentially expressed lncRNA. However, the database lacks information related with *Rattus Norvegicus* specie and the RNA-lncRNA interaction analysis could not be performed.

#### Associated diseases

The association of identified differentially expressed genes with human diseases was analyzed using the Comparative Toxicogenomics Database^10^.

### Statistical analyses

All data were expressed as mean ± standard deviation. The differences among samples were analyzed using Two-Way Repeated Measures ANOVA and Tukey’s test. The value of *p* < 0.05 was considered as statistically significant. Statistical analysis and figuring drawing were carried out using GraphPad Prism 9.0 (GraphPad Software Inc., San Diego, CA, USA). We performed a power analysis on the number of animals using the F test: Fixed effects ANOVA-one way with the free software G*Power V.2.5.

## Results

### Pathological characteristics of diabetic mice

**FIGURE 1:**
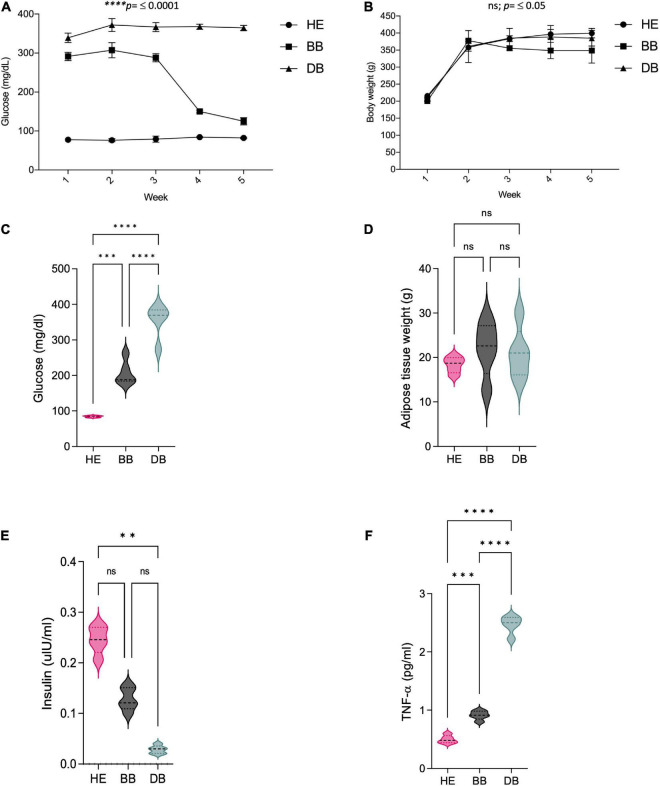
Effects of 5 weeks consumption of an anthocyanin-rich BB extract on **(A)** fasting blood glucose; **(B)** body weight; **(C)** glucose levels at the end of the treatment; **(D)** adipose tissue weight; **(E)** insulin levels; and **(F)** TNF-α levels. Values represent the mean and S.D. (*n* = 8). ^**^p ≤ 0.01; ^***^p ≤ 0.001 and ^*⁣*⁣**^p ≤ 0.0001 when BB were compared to the corresponded value of DB.

After a low dose injection of STZ, rats in BB and DB groups showed significantly increased fasting blood glucose levels compared with animals from HE group, this tendence was observable during the 5 weeks treatment (Figure 1A) and at the end of the treatment as showed in Figure 1C. Furthermore, STZ administration resulted in decreased body weight in BB and DB groups (Figure 1B), but no difference was observed on adipose tissue weight (Figure 1D). These findings demonstrate the success of T2DM induction in Wistar rats.

### Biochemical effects of black beans extract in T2DM rats

Two of the main characteristics of diabetic patients are the body weight decrease and the chronic increase of blood glucose levels. As exposed in Figure 1, the body weight of all the rats increased progressively, and their blood glucose levels were normal. However, the growth rate of the rats in the HE group was slower. Conversely, after STZ administration, the body weight of all rats started to decrease, and their blood glucose levels augmented significantly compared with rats in HE group. Moreover, rats in both the BB and DB groups tended to show a decrease in weight after STZ administration. The effect of BB on the fasting blood glucose level of the rats is shown in Figure 1A, from which it is obvious that the blood glucose levels of rats in the BB group decreased gradually after 1 week of treatment. At the end of treatment period, rats in BB group showed significant (*p* < 0.05) decreases in fasting blood glucose levels. The levels of insulin showed a down trend, even though the decreases were not significant between BB and DB groups (Figure 1E). Further, TNF-α levels in the BB group exhibited a significant decrease (*p* < 0.05) when compared with DB group (Figure 1F).

### Black beans extract significantly modulate global gene expression profile in adipose tissue

**FIGURE 2:**
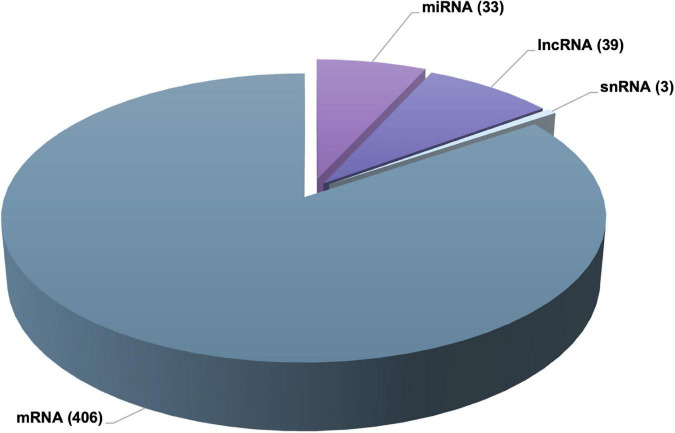
Pie chart of the number of differentially expressed mRNAs, miRNAs, and lncRNAs in diabetic rats treated with anthocyanin-rich black beans extract.

With the aim to identify genomic impact of BB extract on genomic profile in adipose tissue, we performed global RNAseq. Following statistical analysis, we identified 566 significantly differentially expressed genes. A closer understanding into identified differentially expressed genes demonstrated that there are 406 among them that are protein coding genes (mRNAs), 33 are miRNA family genes, 39 are long non-coding genes, 3 are snRNAs and 85 are unidentified (Figure 2). The fold changes of these genes were observed to fluctuate from −1.15 to −13.45 for downregulated genes and from 1.14 to 22.91 for up-regulated genes. These data suggest that the consumption of BB extract can considerably affect the expression of genes, not only protein coding but also protein non-coding genes in adipose tissue. The list of differentially expressed genes were then submitted to functional bioinformatic analyses.

### Functional analysis: Gene ontology, network, and pathway analysis of differentially expressed genes

**FIGURE 3:**
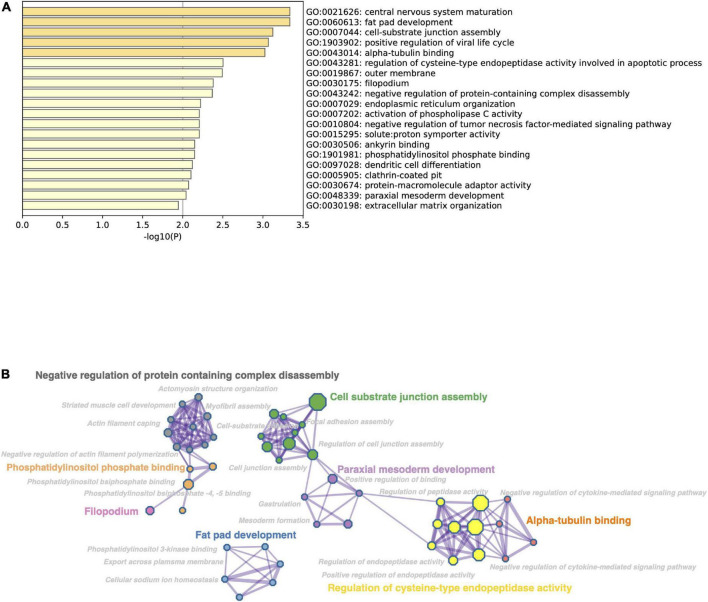
**(A)** Bar graph of enriched gene ontology (GO) terms across input gene list, colored by *p*-values. **(B)** Subset of enriched terms selected to create a network analysis. Terms with a similarity > 0.03 are connected by edges. Bigger nodes represent larger gene sets. The analyzes were performed in Metascape and visualized with the Cytoscape tool.

**FIGURE 4:**
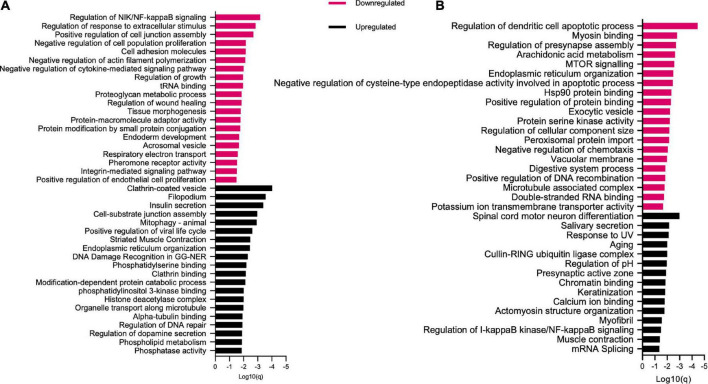
Pathway enrichment analysis from up and downregulated differentially expressed genes in **(A)** BB group (diabetic rats treated with Anthocyanin-rich black beans extract). **(B)** DB group (diabetic rats without treatment).

With the objective to inquire in the cellular functions of significantly moderated protein coding genes, we first conducted gene ontology enrichment analysis using Metascape and Cytoscape tools. Gene ontology analysis by *p*-value indicated that black bean extract impacted numerous biological functional categories that include cell substrate junction assembly, phosphatidylinositol phosphate binding, fat pad development, regulation of cysteine-type endopeptidase activity, among others (Figure 3A). Additionally, we performed a network analysis of over-represented gene ontologies where terms with a *p*-value <0.05, a minimum count of 3, and an enrichment factor >1.5 are collected and grouped into clusters based on their similarities (Figure 3B). To obtain a more detailed understanding of the cellular functions that are regulated by protein coding genes significantly modulated by black bean extract, we then performed pathway enrichment analysis of up- and down-regulated differentially expressed genes using Metascape tool (Figure 4). The results showed that anthocyanin-rich BB extract changed the expression of genes up regulating important pathways in T2DM pathogenesis like insulin secretion, cell-substrate junction assembly, ER organization, phosphatidylserine binding, phosphatidylinositol 3-kinase binding, among others. On the other hand, BB extract also altered the expression of genes that downregulate signaling pathways involved with regulation of NIK/NF-kappaB, regulation of response to extracellular stimulus, positive regulation of cell junction assembly, negative regulation of cell population proliferation, cell adhesion molecules and negative regulation of actin filament polymerization.

**FIGURE 5:**
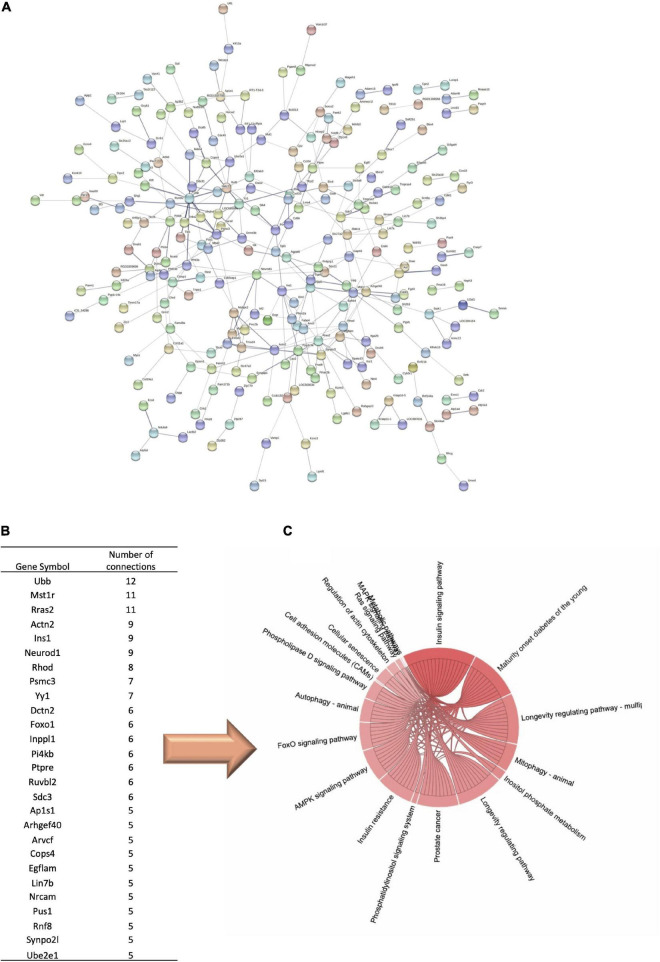
Protein-protein interaction network. **(A)** Interactions between proteins encoded by genes modulated by anthocyanin-rich black beans extract supplementation in diabetic rats. **(B)** Top genes forming major interaction nodes in the network. **(C)** Pathway enrichment analyses of hub proteins encoded by genes.

Our next step was to use STRING database to explore the potential protein–protein interactions of genes identified as differentially expressed by black bean extract intake. The analysis revealed a network of interactions between identified proteins as presented in Figure 5A, as well as genes that form nodes in the network. The next step was to select the genes with the highest number of interactions with other genes and which potentially play an important role in multi-genomic effects. The number of interactions reached 12 for UBB (Ubiquitin B), or 11 for MST1R (macrophage stimulating 1 receptor) and RRAS2 (RAS Related 2), or proteins like INS1 (Insulin-1 precursor) or INPPL1 (Inositol Polyphosphate Phosphatase Like 1) with 5 or more interactions (Figure 5B). Interestingly, pathway enrichment analyses of hub proteins conducted in GeneTrail revealed that these genes are involved in insulin signaling, mature onset of diabetes, insulin resistance, inositol phosphate metabolism or AMPK signaling pathway (Figure 5C).

### Transcription factors potentially involved in the nutrigenomic effect of BB extract

**FIGURE 6:**
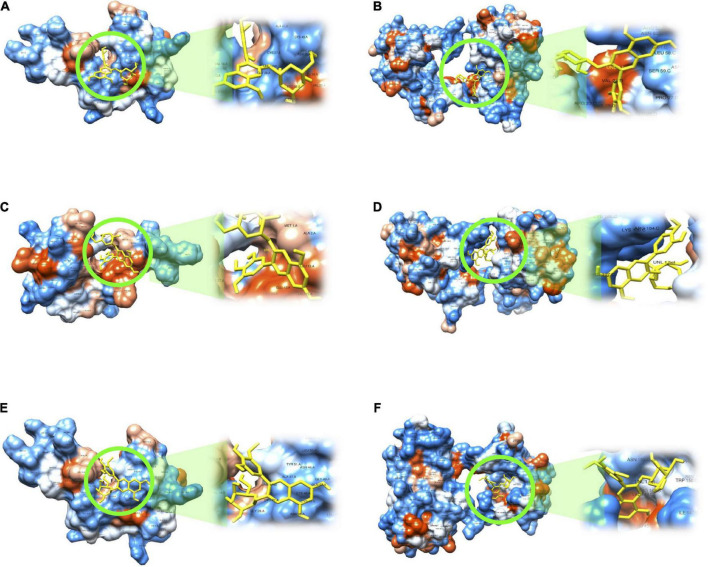
*In silico* docking analysis of interactions between main anthocyanins found in black bean extract and potential transcription factors. **(A)** Delphinidin 3-glucoside to GATA2; **(B)** Delphinidin 3-glucoside to POU2AF1; **(C)** Petunidin 3-glucoside to GATA2; **(D)** Petunidin 3-glucoside to POU2AF1; **(E)** Malvidin 3-glucoside to GATA2; **(F)** Malvidin 3-glucoside to POU2AF1.

Our next objective was to identify transcriptional regulators involved in the observed changes of genes, that is, transcription factors which could have their activity altered by black bean extract and affect the expression of identified significantly modulated genes. To this end, we used the database TRANSFAC and JASPAR using the Enrichr platform. Among the top ten transcription factors identified are GATA2, POU2AF1, IRF3, GATA1, NR2F2 or PPARA (Table 1). It could be suggested that circulating polyphenol metabolites generated after BB extract intake could interact with transcription factors and/or cell signaling proteins regulating their activity. With the aim to test this hypothesis, we searched the capacity of major metabolites of black bean to interact and bind to these proteins using a 3D docking online server. We assessed the binding capacity of 3 major metabolites, delphinidin 3-glucoside, petunidin 3-glucoside and malvidin 3-glucoside: delphinidin 3-glucoside to GATA2 (Figure 6A); delphinidin 3-glucoside to POU2AF1 (Figure 6B); petunidin 3-glucoside to GATA2 (Figure 6C; petunidin 3-glucoside to POU2AF1 (Figure 6D); malvidin 3-glucoside to GATA2 (Figure 6E) and malvidin 3-glucoside to POU2AF1 (Figure 6F). We observed that petunidin 3-glucoside showed potential binding capacity of -6.4 kcal/mol to POU2AF1, as well as petunidin 3-glucoside and delphinidin 3-glucoside with GATA2 (−6.2 kcal/mol), and POU2AF1 (−6.2 kcal/mol), respectively. These results shows that anthocyanins in BB can interact with cell signaling proteins and produce changes in their kinase activity, this modulates the activity of downstream cell signaling proteins and consequently transcription factors, The described changes could result in the observed gene expression modifications.

**TABLE 1:** Potential transcription factors that modulate differentially expressed protein coding genes with consumption of anthocyanin-rich black beans extract.

Symbol	Name
GATA2	GATA binding protein 2
POU2AF1	POU Class 2 Homeobox Associating Factor 1
SIX1	SIX Homeobox 1
BCL3	BCL3 Transcription Coactivator
REST	RE1 Silencing Transcription Factor
IRF3	Interferon regulatory factor 3
MECP2	methyl CpG binding protein 2
GATA1	GATA Binding Protein 1
NR2F2	nuclear receptor subfamily 2 group F member
PPARA	peroxisome proliferator activated receptor alpha

### miRNA – Identification of their targets and functional analyses

**FIGURE 7:**
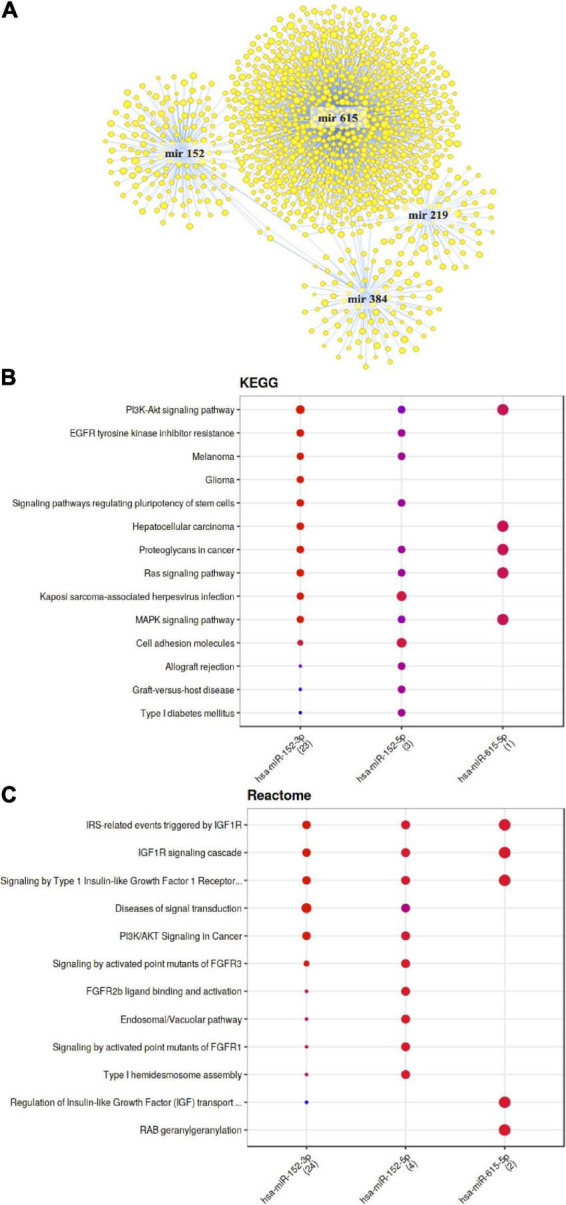
Bioinformatic analyses of differentially expressed miRNA and their target mRNA. **(A)** Network of miRNA significantly modulated with consumption of anthocyanin-rich black beans extract and their target mRNA. **(B)** KEGG pathways associated with miRNA modulated with consumption of anthocyanin-rich black beans extract. **(C)** Reactome pathways associated with miRNA modulated with consumption of anthocyanin-rich black beans extract.

Our gene expression analysis also allowed us to surmise that BB can also lead to alterations in the expression of not only protein coding RNAs but also non-coding RNAs, such as miRNAs. We observed changes in expression of 33 miRNAs, including Mir615, Mir152, Mir219a1 or Mir384. Using existing database, we searched for target genes of the identified miRNAs, and nearly 500 target genes were identified. These target genes and identified miRNAs form a network of interactions as presented in the Figure 7A. To identify potential cellular functions affected by these miRNAs, we cross-examined the Mienturnet database to reveal over-represented pathways from both KEGG (Figure 7B) and Reactome (Figure 7C) databases, that is pathways associated with each of the miRNA recognized as differentially expressed by black bean extract. Among the pathways identified are PI3K- signaling pathway, Ras signaling pathway, type 1 diabetes mellitus, Insulin receptor substrate 1 related pathway, and regulation of insulin-like growth factor.

### IncRNA modulated by anthocyanin-rich black beans extract

As we mentioned in the materials and methods section, we were not able to perform the RNA-lncRNAs interaction analysis in LncRRIresearch web server. However, in Supplementary Figure 1 we show the list of 39 lncRNAs with their fold changes that were modulated by the anthocyanin-rich BB extract.

### Correlation with diseases

Together with identification of cellular mechanisms affected by different types of RNAs, we also aimed to identify diseases associated with identified differentially expressed genes. We used the Enrichr database OMIM disease tool that interconnects differentially expressed genes with diseases, revealing their possible role in prevention or development of these disorders. We observed that our genes differentially expressed between the BB and DB groups are significantly associated with metabolic disease, nutrition disorder, cardiovascular disease, and immune system disease (Table 2).

**TABLE 2:** Associations of gene expression profile of consumption of anthocyanin-rich black beans extract with known human diseases.

Disease name	Disease ID	Disease categories	*P*-value	Annotated genes number
Metabolic diseases	MESH:D008659	Metabolic disease	0.01568	21
Metabolism, inborn errors	MESH:D008661		0.03066	11
Cardiovascular diseases	MESH:D002318	Cardiovascular disease	2.92E-06	31
Heart diseases	MESH:D006331		1.23E-04	22
Vascular diseases	MESH:D014652		1.46E-04	20
Cardiovascular abnormalities	MESH:D018376		0.00967	6
Cerebrovascular disorders	MESH:D002561		0.03663	5
Heart defects, congenital	MESH:D006330		0.00636	6
Heart septal defects	MESH:D006343		0.02307	2
Hypertension	MESH:D006973		0.01541	6
Myocardial ischemia	MESH:D017202		0.00904	9
Myocardial reperfusion injury	MESH:D015428		0.03506	3
Reperfusion injury	MESH:D015427		0.01257	5
Stroke	MESH:D020521		0.04584	3
Thromboembolism	MESH:D013923		0.01362	2
Immune system diseases	MESH:D007154	Immune system disease	1.87E-04	23
Hydrops fetalis	MESH:D015160		9.64E-04	2
Erythroblastosis, fetal	MESH:D004899		0.00134	2
Arthritis, juvenile	MESH:D001171		0.02347	4
Autoimmune diseases	MESH:D001327		0.00589	11
Dermatitis, atopic	MESH:D003876		0.03994	2
Nutrition disorders	MESH:D009748	Nutrition disorder	0.00754	7
Obesity	MESH:D009765		0.00987	6
Overnutrition	MESH:D044343		0.00987	6
Overweight	MESH:D050177		0.00987	6
Pathologic processes	MESH:D010335	Pathologic alterations	5.32E-07	45
Fibrosis	MESH:D005355		2.99E-04	20
Liver cirrhosis, experimental	MESH:D008106		7.07E-04	16
Liver cirrhosis	MESH:D008103		0.0012	17
Hyperuricemia	MESH:D033461		0.00411	2
Myocardial reperfusion injury	MESH:D015428		0.03506	3
Reperfusion injury	MESH:D015427		0.01257	5

## Discussion

**FIGURE 8:**
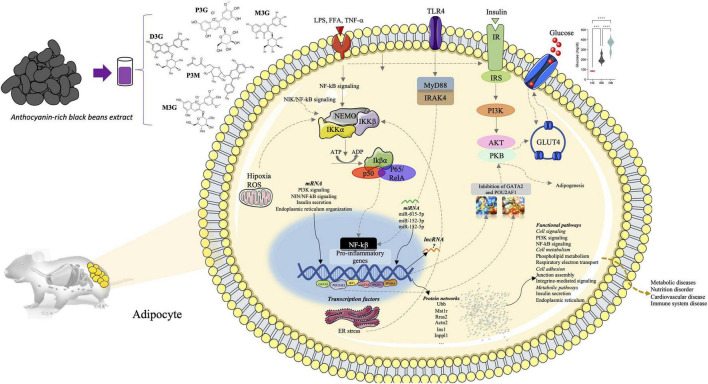
Summary figure representing multi-omic results and genomic modifications induced in diabetic rats by anthocyanin-rich black beans extract supplementation.

In this research we investigated the potential health benefits and the multigenomic mode of action of a rich-anthocyanin extract from BB on adipose tissue in the context of diet-streptozocin-induced type 2 diabetes mellitus. After 4 weeks dietary supplementation, we found that black bean extract improved the symptoms of T2DM and insulin resistance, controlled the levels of blood glucose, and pro-inflammatory cytokines. The use of RNAseq revealed a complex multigenomic mode action of these bioactive compounds in adipose tissue by modulating expression of protein coding, miRNA, transcription factors, and lncRNAs (Figure 8), regulating processes like inflammation, metabolism and cell signaling.

In the last years, special interest was given to research on natural and non-toxic antidiabetic agents. Plants are important sources of bioactive compounds with numerous valuable health effects. Functional foods contain bioactive compounds that can exert health advantages beyond their natural properties when consumed in a regular and consistent manner through diet (32). Anthocyanins are an important class of polyphenols featured by their promising effects on T2DM acting on (a) suppression of carbohydrate-metabolizing enzymes; (b) decrease of glucose transporters expression or activity; (c) inhibition of glycogenolysis and (d) modifying the gut microbiota by anthocyanin breakdown products (33). In this research we found that an anthocyanin-rich BB extract improved glucose levels on diabetic rats. One of the effects of anthocyanins in T2DM is the suppression of postprandial glycaemia through the inhibition of α-amylase and α-glucosidase enzymes. In a study conducted by Törrönen et al., the authors evaluated the effect of berries, naturally rich in anthocyanins, on postprandial glucose levels in healthy volunteer adults. Another study showed that consumption of a rich-anthocyanin puree containing bilberries, blackcurrants, cranberries, strawberries, and 35 g of sucrose resulted, after 15 and 30 min, in lower levels of glucose when compared with the control group that only consumed sucrose (34). Using *in silico* and *in vivo* studies, pelargonidin-3-*O*-rutinose present in strawberries exhibited the potential to improve postprandial hyperglycemia by inhibiting α-glucosidase (35). Another mechanism of action of anthocyanins is their impact on glucose transporters. It was demonstrated that an anthocyanin-rich berry-extract considerably decreased sodium-dependent and sodium-independent transporters in Caco-2 cells and also reduced the expression of genes encoding SGLT1 and GLUT2, suggesting that anthocyanins can regulate the rate of glucose absorption (36). The over-expression of glycogenolysis in the liver releases glucose into the bloodstream; glycogen synthase kinase (GSK3β) is a key liver enzyme that inhibits glycogen synthase (GS) enzyme to convert glycogen to glucose. Herrera-Balandrano et al., investigated the hypoglycemic effects of malvidin from a blueberry anthocyanin extract (BAE) and observed that BAE could improve insulin sensitivity by inhibiting GSK3β and glycogen synthase in the insulin-independent pathway (37). Moreover, a recent systematic review describes that anthocyanins can also exert their health effects by modulating the gut microbiota composition, particularly by increasing *Bacteroidetes* and decreasing *Firmicutes*. These changes will result in higher production of short chain fatty acids, lower intestinal permeability and pH, greater number of goblet cells and improvement of villi anatomy (38). A study conducted on diabetic Zucker rats supplemented with an anthocyanin-rich extract from purple potato and bilberry, showed a reduction in dysbiosis of colonic microbiota and anthocyanins gut microbiota-derived metabolites, increased cecal sugar levels together with the increase in the abundance of *Peptostreptococcaceae* sp. and *Parabacteroides* spp. in colon. These results demonstrate that anthocyanins by modulating the gut microbiota can affect gut function and consequently prevent T2DM (39).

Our RNAseq analysis showed for the first time that an anthocyanin-rich BB extract can affect the expression of large number of genes in adipose tissue, not only protein coding but also non-coding genes like miRNAs or lncRNAs. According to our knowledge, very few studies have reported the multigenomic effect of BB anthocyanins, as well as isolated anthocyanins, on adipose tissue. It has been observed that cyanidin 3-glucoside, anthocyanin from BB, can up-regulate the expression of GLUT4 gene *in vitro* in 3T3-L1 cells (40). The BB extract rich in flavonoids and saponins was also shown to exert the capacity to modulate the expression of key genes involved in lipogenesis, such as SREBP1c, ABCG5, CPT1, SREBP2, FAS, HMGCR, INSIG1 and INSIG2 (41). Supplementation of mice diet with BB has been reported to modulate the expression of the SCFA G-protein coupled receptors (GPR-43, GPR109a, GPR-41) in colonic tissue as well as the expression of genes regulated the integrity of the epithelial barrier: occludin, JAM-A, E-cadherin and the junction/cytoskeletal connector ZO-1 (42). Recently it has been observed that black bean concentrate improves hepatic steatosis by reducing lipogenesis and increasing fatty acid oxidation in rats fed a high fat-sucrose diet (43). However, these studies present an important drawback, which is use of targeted approach to assess the expression of few specific genes. Rare recent studies have shown that anthocyanins can affect large number of genes presenting multi-modal action. For example, anthocyanins from blackberries affected the expression of over 600 genes, including miRNAs, in circulating immune cells in human volunteers (44). Another study has demonstrated the capacity of anthocyanin-rich bilberry extract to modulate the expression of over 1,600 genes in hippocampus of ApoE-/- mice (45).

Enrichment pathway analysis in adipose tissue were performed using 406 differentially expressed genes following BB extract intake. This allowed us to show that the black bean extract can affect genes regulating cell signaling pathways in adipose tissue from diabetic rats, pathways related with phosphatase activity, phospholipid metabolism, phosphatidyl inositol 3-kinase binding, phosphatidylserine binding, and ER organization. On the other hand, we also found down-regulated pathways that participate in the modulation of NIK/NF-κB signaling, regulation of response to extracellular stimulus, positive regulation of cell junction assembly, negative regulation of cell population proliferation and negative regulation of cytokine-mediated signaling pathway. Remarkably, while regulation of NIK/NF-κB signaling is up-regulated in the DB group, we observed that the treatment with BB extract down-regulated this pathway in the BB group, suggesting the capacity of the extract to counteract the effect of T2D. The biological relevance of this pathway on adipose tissue is the critical function in reprogramming the fat cell transcriptome to inflammation in response to overnutrition and metabolic stress. The inhibition of NF-κB signaling has metabolic advantages for adipose tissue inflammation caused by obesity (46). On the other hand, NF-κB inducing kinase (NIK) is a key controller of immunity and inflammation at local and systemic levels on metabolic processes. In adipose tissue, NIK promotes adipogenesis by activating non-canonical NF-κB pathway, demonstrated by Pflug et al., when NIK deficient mice fed with a high fat diet showed decreased overall fat mass, increased insulin sensitivity, and energy expenditure (47). Moreover, anti-inflammatory effects have also been attributed to anthocyanins from *Hibiscus syriacus* L. by the capacity to inhibit TLR4 in LPS-induced cells, followed by the decrease of the phosphorylation of MyD88 and IRAK4, which resulted in NF-κB inactivation (48). Black soybeans also demonstrated to inhibit IκB phosphorylation that impede NF-κB translocation resulting in the inhibition of iNOS transcription and iNOS and COX-2 translation (49). Taken together, these results suggest that extract from BB can exert health properties by countering the genomic modifications induced by T2DM.

Elevated levels of FFAs, inflammation, excess of nutrients, inadequately folded proteins, and local hypoxia, are characteristics of obesity and can lead to ER stress. This results in increased oxidative stress in adipose tissue of obese animals (50). ER organization pathway was positively regulated in BB group but not in DB group. ER plays an important role in adipose tissue inflammation due to their participation in the activation of NF-κB signaling by the phosphorylation of JNK, IKK, and JNK-mediated phosphorylation of IRS1/2 triggering the unfolded protein reaction (UPR) and involving pathways like PERK (PKR-like ER kinase), IRE1 (requiring enzyme 1), and ATF6 (activating transcription factor 6) (51). Exposure of J774A.1 macrophages to a cyanidin-3-O-galactoside-rich aqueous extract of *Sambucus ebulus* L. (SE) resulted in suppression of LPS-induced transcription of pro-inflammatory genes like *IL-1β, IL-6, TNF-α, Ccl2, Icam-1, Fabp4, COX2, iNOS, Noxo1, IL-1ra and Sirt1.* SE also showed to produce the decrease in protein levels of iNOS, peIFα, ATFα and C/EBP Homologous Protein (CHOP) (50). Similarly, *Punica granatum* L. a high-content polyphenols Chinese plant, (PGF) was administrated to diabetic rats for 4 weeks. It was observed that doses between 50 and 100 mg/kg can improve ER stress signals including IRE1, activation of XBP-1, as well as lower levels of IREα, XBPs, and CHOP (52). Therefore, anthocyanins present in our extract also have the capacity to modulate the expression of genes regulating ER stress, presenting another important molecular target underlying the anti-diabetic effects. Our bioinformatic analysis also revealed that phosphatidylinositol 3-kinase binding signaling pathway was up-regulated in BB group. The importance of this pathway in adipose tissue is due to its role in adipose hypertrophy which rises tissue immune cell infiltration, fibrosis, and lipolysis. Decreases IRS-1 activation and AKT-induced glucose uptake, as well as exacerbates systemic insulin resistance and the development of T2DM. Different factors like adipocytokines and adipose hypertrophy produce insulin resistance by obstructing PI3K/AKT-mediated inhibition of lipolysis reducing the capacity of glucose utilization and diminishing the capacity of SREBP to stimulate lipid synthesis (53). Recent research concluded that sweet potato leaf polyphenols might up-regulate the important mediators of the insulin-mediated PI3K/AKT/GSK-3β signaling pathway in a dose-dependent way in diabetic mice by regulating the mRNA expression of genes IR, IRS-1, PI3K, AKT and GLUT-4 (54).

Bioinformatic analysis of differentially expressed genes permitted to identify potential transcription factors whose activity can be affected by BB extract and result in the detected nutrigenomic modifications. Among these transcription factors are GATA2, POU2AF1, IRF3, GATA1, NR2F2 or PPARA. Interestingly some of these transcription factors have been identified as playing a role in adipose tissue development and/or diabetes. For example, it has been shown that GATA2 plays an important role in diabetes development and associated diseases (55). It has been described that hyperinsulinemia, observed during Type 2 diabetes, can activate NR2F2 which can induce development of different diseases (56). PPARs have central role in lipid and glucose homeostasis, pathogenesis of insulin resistance and metabolic syndromes and are active in adipose tissue. It has been shown that binding phenolic and other molecules to PPARs result in significant changes in their activities and present important mode of treatment type 2 diabetes mellitus. (57). Similarly, IRF3 has been described as a main transcriptional regulator of adipose tissue inflammation and is involved in preserving systemic glucose and energy homeostasis (58). One study also observed that IRF3 in adipose tissue promotes adipose inflammation and insulin resistance (58). These observations from our bioinformatic analysis reveal significant regulators of BB extract underlying their observed health properties.

Moreover, our genomic analysis revealed that black bean extract rich in anthocyanins can also modulate the expression of microRNAs. MicroRNAs (miRNAs) are short, non-coding RNAs which can bind to mRNAs, resulting in modification in translational repression. It is estimated that there are approximately 2200 miRNA genes in the human genome that can regulate the transcriptional levels of over 60% of genes. Consequently, they can regulate many major cellular functions such as development, differentiation, growth, or metabolism (59). Moreover, it has been shown that modifications in their expression produce an important role in progress of diseases. Among these, it has been demonstrated that miRNA might have an important role in development of diabetes and metabolic disorders (60, 61). On the other hand, several previous studies have shown that anthocyanins can modulate the expression of these non-coding RNAs. For example, it was shown that a mixture of anthocyanins as well as their gut microbiome derived metabolites at physiologically relevant concentrations can affect the expression of miRNAs in isolated human primary endothelial cells (62). Also, a study showed that supplementation of mice diets with different polyphenols at nutritionally relevant doses can significantly affect global miRNA expression profile in liver (63). Among the miRNAs identified as differentially expressed by black bean extract are miR-152, miR-219a1, miR-384 or miR-615. It has been observed that miR-152 is expressed in adipocytes and can stimulate lipid accumulation in preadipocytes accompanied by higher expression of some pro-adipogenic genes, adipogenesis and intramuscular fat formation (64). Its expression is also altered in patients with T2D with or without medication (65). miR-219a1 has been suggested to show a role in fat development. Similarly, it has been demonstrated that miR-615 is expressed in adipose tissue in humans and that its expression changes with the obesity (66). Moreover, the expression of this miRNA has also been identified in patients with diabetes (67). Together with miRNAs, our analysis also revealed changes in the expression of long non-coding RNAs. Biogenesis of lncRNAs is different than mRNAs and can interact with DNA, RNA, and proteins, modulating chromatin function, changing the stability and translation of cytoplasmic mRNAs and interfering with signaling pathways (68). Because of their large mode of action, they affect numerous cellular and consequently physio-pathological processes. It has been shown that they regulate adipogenesis and adipose tissue function (69) and diabetes (70). Only few studies have suggested that polyphenols are potent modulators of the expression of non-coding RNAs. For example, epicatechin metabolites can regulate the expression of these miRNAs in human brain endothelial cells in physiologically relevant conditions (31). Therefore, our study provides novel and original data about the capacity of legumes and their bioactives to affect the expression of this class of RNAs. However due to the lack of known biological functions of these lncRNAs as well as their targets, regulated cellular and molecular processes remain to be identified. Taken together, our results suggest that black bean extract rich in anthocyanins exert protective properties by modulating the expression of miRNAs and lncRNAs in adipose tissue in diabetic conditions.

## Conclusion

Our multi-genomic approach including transcriptomic, microRNomic, lncRNomic and proteomic revealed the regulation of a rich-anthocyanins black bean extract on adipose tissue in diabetic rats, demonstrating that these metabolites can regulate a large number of interactions involved in phospholipid metabolism, phosphatidyl inositol 3-kinase binding, phosphatidylserine binding and ER organization, and NIK/NF-κB pathways. In agreement with our hypothesis, we can argue that gene expression profile in adipose tissue from diabetic rats exposed to BB extract is negatively correlated with the expression of genes observed in diabetic rats. The observed alterations in gene expression in this study might be a link to the protective anti-diabetic effects of BB in other metabolic diseases. Future research is needed using this multi-omics approach including not only genomic but also studies of health effects.

## Data availability statement

All RNAseq data are available at GEO database under accession number GSE215903 (https://www.ncbi.nlm.nih.gov/geo/query/acc.cgi?acc=GSE215903).

## Ethics statement

The animal study was reviewed and approved by Internal Committee of Care and Use of Laboratory Animals (CICUAL) of the Center for Research and Assistance in Technology and Design of the State of Jalisco, A.C., (CIATEJ A.C.).

## Author contributions

EL-C supported with funding acquisition, supervision, and project administration. EL-C, KD-M, YS-M, LF-Y, and EM-M contributed to the conception, study design, and edited the manuscript. KD-M, DM, and KC-J carried out experimental work, performed data analysis, interpretated the results, and edited the manuscript. All authors contributed equally and approved the final version of the manuscript.

## Footnotes

1
http://bioinformatics.sdstate.edu/go

2
https://cytoscape.org

3
https://metascape.org/gp/index.html#/main/step1

4
https://string-db.org/

5
http://userver.bio.uniroma1.it/apps/mienturnet/

6
https://amp.pharm.mssm.edu/Enrichr/

7
http://www.swissdock.ch/docking

8
https://www.uniprot.org

9
https://pubchem.ncbi.nlm.nih.gov

10
https://ctdbase.org/

## Supplementary material

Supplementary Figure 1 List of lncRNA and their fold changes modulated by anthocyanin-rich black beans extract.
