# Supplementary figures and images for: Anthocyanin-rich extract from black beans exerts anti-diabetic effects in rats through a multi-genomic mode of action in adipose tissue
Source: Front Nutr. 2022 Nov 14;9:1019259. doi: 10.3389/fnut.2022.1019259 (PMC9702351; doi:10.3389/fnut.2022.1019259)

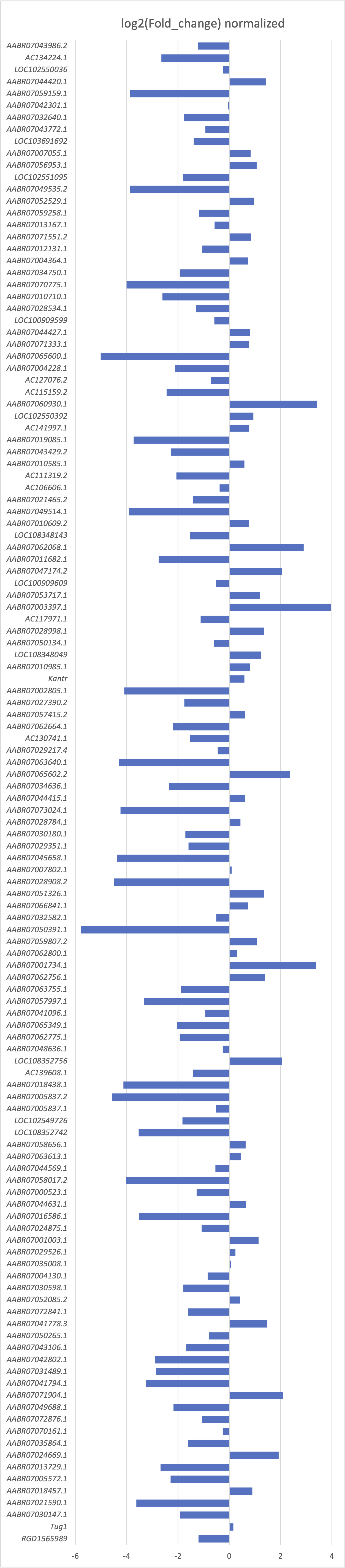

Supplement: Supplementary Figure 1 — List of lncRNA and their fold changes modulated by anthocyanin-rich black beans extract. [file Image_1.TIFF]
